# Analytical, toxicological and kinetic investigation of decomposition of the drug diclofenac in waters and wastes using gamma radiation

**DOI:** 10.1007/s11356-015-5236-6

**Published:** 2015-08-27

**Authors:** A. Bojanowska-Czajka, G. Kciuk, M. Gumiela, S. Borowiecka, G. Nałęcz-Jawecki, A. Koc, J. F. Garcia-Reyes, D. Solpan Ozbay, M. Trojanowicz

**Affiliations:** Institute of Nuclear Chemistry and Technology, Dorodna 16, 03-195 Warsaw, Poland; Department of Chemistry, University of Warsaw, Pasteura 1, 02-093 Warsaw, Poland; Department of Environmental Health Sciences, Medical University of Warsaw, Warsaw, Poland; Faculty of Physical and Analytical Chemistry, University of Jaen, Jaen, Spain; Department of Chemistry, Hacettepe University, Ankara, Turkey

**Keywords:** Diclofenac, Radiolytic decomposition, Pulse radiolysis, Toxicity monitoring

## Abstract

The radiolytic decomposition of the drug diclofenac (DCF), and in limited extent, also two other widely used drugs, ibuprofen and carbamazepine, was examined using liquid chromatography (LC) methods. The efficiency of DCF decomposition was examined in function of the absorbed dose of gamma radiation, and also in the presence of selected scavengers of radicals, which are commonly present in natural waters and wastes. Three different tests were employed for the monitoring of toxicity changes in the irradiated DCF solutions. The LC/mass spectrometry (MS) was used for the determination of products of DCF radiolysis. Using pulse-radiolysis method with the spectrophotometric detection, the rate constant values were determined for reactions of DCF with the main products of water radiolysis: hydroxyl radicals (1.24 ± 0.02) × 10^10^ M^−1^ s^−1^ and hydrated electrons (3.1 ± 0.2) × 10^9^ M^−1^ s^−1^. Their values indicate that both oxidative and reductive processes in radiolytic decomposition of DCF can take place in irradiated diluted aqueous solutions of DCF. The possibility of decomposition of all examined analytes was investigated in samples of river water and hospital waste. Compared to the previous studies, the conducted measurements in real samples were carried out at the concentration levels, which are close to those reported earlier in environmental samples.

**Graphical abstract:**
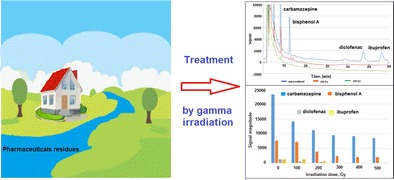
ᅟ

ᅟ

## Introduction

The wide use of pharmaceuticals by the contemporary society unfortunately results in an important side effect, which is an increasing danger for the natural environment by pharmaceutical residues. The first reports about detecting pharmaceuticals in environmental samples were published in the early 1970s [Tabak et al. 1970], and in 1965, it was observed for the first time that residues of steroid hormones are not completely decomposed by wastewater treatment [Stumm-Zollinger, 1965]. Since then, a fast increase of interest in different aspects of the presence of pharmaceutical residues in environmental waters is observed. Number of papers published annually was about 500 in 2000, while it reached the level of about 3000 in 2010 [Fatta-Kassinos et al. 2011]. In the recent decade, this problem was a subject of several published books, e.g., [Kümmerer 2008], and numerous valuable review articles in scientific journals, e.g., [Fatta-Kassinos et al. 2011, Mompelat et al. 2009, Luo et al. 2014].

A common presence of pharmaceutical residues in the aquatic environment, and also in finished drinking water, is a source of concern about their impact on public health, although a commonly encountered opinion in the literature is that our current knowledge about the effects of low-dose mixture of pharmaceuticals on human health is very limited. One can find opinions about no appreciable risk to humans at detected concentrations of pharmaceutical residues [Schulman et al. 2002], but due to consuming the contaminated drinking water over a lifetime, chronic toxic effects cannot be excluded because of a lack of chronic ecotoxicity data [Carlsson et al. 2006].

Pharmaceuticals disposed to the natural environment are very large and diverse group of chemicals consisting of both human and veterinary medicinal compounds. The main groups of pharmaceuticals, which are detected in aqueous environment include anti-inflammatory drugs (analgestics), steroids and related hormones, antibiotics, β-blockers, and lipid regulators. Some of them are consumed annually even in tens or hundreds of tons. For instance, the non-steroidal anti-inflammatory drug paracetamol, 622 t in Germany in 2001; ibuprofen, 345 t in Germany in 2001; diclofenac, 86 t in Germany in 2001; and naproxen 35 t in England in 2001 [Fent et al. 2006]. The trend of the continuous increase of consumption of pharmaceuticals can be illustrated e.g., for data on antidepressants consumption from 2000 to 2010 (OECD 2012). In all European countries for which data is available, the consumption of antidepressants has increased by over 80 % on average across EU member states. In another example, the diclofenac consumption in Netherlands was evaluated as 2124 μg/cap/day in 2012, while in Germany already in 2003 as 2613 μg/cap/day [Johnson et al. 2013].

As the main source of a wide presence of pharmaceuticals in the environment is considered, the municipal water discharge, of which drug residues are not completely removed by current wastewater processes. Other contributing sources are industries, farms and hospitals, although for instance, it was demonstrated recently by studies carried out in Norway that the point sources discharges from hospitals typically make a small contribution to the overall pharmaceutical load in comparison to municipal sources [Langford et al. 2009]. One of the most frequently detected pharmaceuticals in waters and urban wastes is diclofenac (DCF), which is 2-[(2,6-dichlorophenylo)amino]benzene acetic acid, used mostly in form of sodium salt or methyl ester.
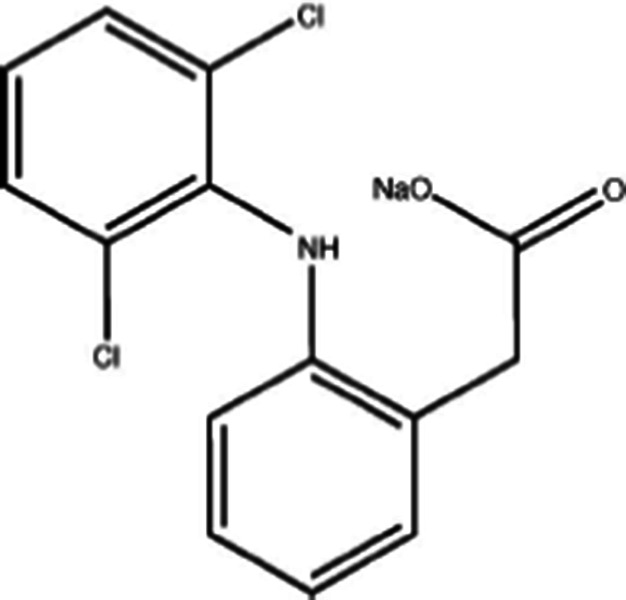


It is widely used in medical care as an analgestic, antiarthritic, and antirheumatic compound, belonging to the group of the non-steroidal anti-inflammatory drugs. Some 15 % of DCF, however, is excreted unchanged after human uptake. It is used worldwide and has a production volume estimated to be in the hundreds of tons annually [Buser et al. 1998]. Among some ecotoxicological effects of the common presence of DCF in the environment, one can indicate e.g., histopathological alterations and bioaccumulation in fishes [Schwaiger et al. 2004], or a discovered vulture population decline in Pakistan [Oaks et al. 2004].

It is estimated that more than 90 % of DCF is eliminated from waters by the photolytic degradation by natural sunlight; however, in recently proposed ranking system for all detected pharmaceuticals, the personal care products and endocrine disrupting chemicals in USA in surface and finished drinking waters, DCF was in 47th position in terms of overall score, and 48th position in terms of ecological effects [Kumar et al. 2010]. In the review on the environmental occurrence of pharmaceuticals, one can find that its content in surface waters may reach even 1.2 μg/L, and in effluents 2.1 μg/L [Daughton et al. 1999], but also the level as high as 5.45 μg/L was reported in some effluents [Ferrari et al. 2003]. Concentration of some pharmaceuticals detected in effluents (antibiotics, non-steroidal anti-inflammatory drugs, or steroid hormones) may reach even fractions of milligrams per liter [Ikehata et al. 2006].

Besides increasing consumption of pharmaceuticals, a significant factor contributing to their presence in the environment is a limited efficiency of their decomposition in wastewater treatment plants, and in drinking water treatment plants. Data recently collected in the above-mentioned review [Luo et al. 2014], indicate only about 35 % removal efficiency of diclofenac in the conventional wastewater treatment plants. This limited efficiency concerns also such methods as UV irradiation, oxidation with free chlorine, or even ozonation [Snyder 2008]. This is then reflected by concentrations of pharmaceuticals and their metabolites in worldwide tap water, which are found in some cases in the level exceeding 1 μg/L [Jones et al. 2005]. Hence, a strong attention is focused in recent years on the development of efficient radical methods of decomposition, described as advanced oxidation processes (AOPs). Especially efficient process is the radiolytic decomposition by the use of ionizing radiation (γ or beam of accelerated electrons, EB), where radicals of oxidative and reductive properties are formed as the result of the water radiolysis taking place during irradiation of a diluted aqueous solutions. These processes were already examined, e.g., for satisfactory decomposition of β-blockers [Song et al. 2008] and antibiotic nitroimidazoles [Sanchez-Polo et al. 2009].

In recent decade, a large attention is focused also on the development of various methods for removal of diclofenac from waters and wastes (Table 1), while effectiveness of removal of DCF in wastewater treatment plants was discussed in a separate review [Zhang et al. 2008]. Data showed in Table 1 indicate that majority of reported AOPs for the decomposition of DCF at mg/L concentration level require relatively a long period of time. The exceptions are ozonation methods, although some essential differences can be found in reported results [Vogna et al. 2004, Naddeo et al. 2009, Zwiener et al. 2000]. Some evident progress in this field was made in the recent few years by the application of such treatment methods as the photocatalytic treatment with TiO_2_ functionalized carbon nanotubes in the presence of H_2_O_2_ [Martinez et al. 2011], the pyrite catalyzed Fenton reaction [Bae et al. 2013], the photocatalytic ozonation [Aguinaco et al. 2012], the sonoelectrochemical treatment [Finkbeiner et al. 2015], or employing the pulsed corona discharge [Dobrin et al. 2013].

**Table 1:** Examples of the application of advanced oxidation processes and electro-oxidation for the decomposition of diclofenac

Method	Initial DCF concentration, mg/L	Efficiency of DCF decomposition	Reference
Photocatalytic, TiO_2_	15	Complete in 1 h	[Calza et al. 2006]
	5–80	Complete 5 mg/L in 1 h	[Rizzo et al. 2009]
	10	61 % in 1 h	[Achilleos et al. 2010]
Photocatalytic with H_2_O_2_	296	90 % in 90 min	[Vogna et al. 2004]
Photocataltytic with TiO_2_ functionalized CNT and H_2_O_2_	8.0	Complete in 10 min	[Martinez et al. 2011]
Solar radiation	50	83 % in 1 day	[Bartels et al. 2007]
Solar radiation	45.5	70 % in 30 min; complete in 160 h	[Aguera et al. 2005]
Solar radiation, TiO_2_	43	Complete in 3 h	[Perez-Estrada et al. 2005a]
Solar photo-Fenton	55	Complete in 100 min	[Perez-Estrada et al. 2005b]
Photo-Fenton	18	Complete mineralization in 1 h	[Ravina et al. 2002]
Pyrite catalyzed Fenton	5.0	Complete in 2 min	[Bae et al. 2013]
Ozonation	296	Complete in 8 min	[Vogna et al. 2004]
Ozonation	40	90 % in 40 min	[Naddeo et al. 2009]
Ozonation, H_2_O_2_	0.002	97 % in 10 min	[Zwiener et al. 2000]
Photocatalytic ozonation	30	Complete in 3 min	[Aguinaco et al. 2012]
Sonolysis	80	55 % in 1 h	[Naddeo et al. 2009]
Sonolysis	40	50 % in 1 h	[Naddeo et al. 2010]
Sonolysis	15	26 % in 1 h	[Nie et al. 2014]
Sonolysis with TiO_2_	50	84 % in 30 min	[Hartmann et al. 2008]
Sonoelectrochemical	0.05	90 % in 5 min	[Finkbeiner e tal. 2015]
Sonophotolysis	10	96 % in 2 h	[Michael et al. 2014]
Catalytic oxidation, H_2_O_2_	20	Complete in 30 min	[Hoffmann et al. 2007]
Oxidation with ClO_2_	0.9	75 % in 1 min	[Wang et al. 2015]
Electro-oxidation	30	72 % mineralization in 4 h	[Zhao et al. 2009]
Pulsed corona discharge	50	Complete in 15 min	[Dobrin et al. 2013]

Recently, first papers on radiolytic decomposition of diclofenac were also published [Liu et al. 2011, Homolok et al. 2011, Kimura et al. 2012, Trojanowicz et al. 2012, Yu et al. 2013]. In studies carried out at initial concentration levels 20–50 mg/L, it was showed that degradation of DCF by γ-irradiation in aqueous solutions can result both from the radical reaction with hydroxyl radical ^•^OH and hydrated electron e_aq_^−^, which are produced from the radiolysis of water [Liu et al. 2011, Homolok et al. 2011, Yu et al. 2013]. It was also indicated that the yield of degradation is larger in acidic medium than neutral or alkaline [Liu et al. 2011]. The changes of Microtox® toxicity were also examined in terms of absorbed radiation dose [Homolok et al. 2011, Yu et al. 2013]. The rate constant for reaction of DCF with ^•^OH was determined as 9.0 [Kimura et al. 2012] and 9.29 × 10^9^ M^−1^ s^−1^ [Yu et al. 2013]. Very recently, when this study was in progress also, the rate constant for the reaction with *e*^−^_aq_ 1.53 × 10^9^ M^−1^ s^−1^ was reported [Yu et al. 2013]. In our initial studies, we also demonstrated the oxidative and reductive pathways of radical degradation of DCF, although a more efficient degradation of DCF with ^•^OH radicals was observed at low absorbed radiation doses, where no complete decomposition is obtained at the initial DCF concentration 50 mg/L [Trojanowicz et al. 2012]. For the identification of main products of DCF decomposition, the gas chromatography-mass spectrometry (GC/MS) and high-performance liquid chromatography (HPLC) with UV detection were employed.

The aim of this work was investigation of the yield of the radiolytic decomposition of DCF by means of gamma radiation, the determination of rate constants for radical reactions of DCF with products of water radiolysis, and also the monitoring of toxicity changes during radiolytic decomposition. For some comparison and more general picture of carried out processes, irradiated solutions contained also two other very commonly used pharmaceuticals—carbamazepine and ibuprofen, and also a very common industrial pollutant bisphenol A. For instance, the presence of carbamazepine was reported in 40 % of large collection of waters intended for human consumption [Houeto et al. 2012], while commonly found ibuprofen in environmental waters may reach in wastewater the concentration level 373 μg/L, and in effluent from wastewater treatment plants 48 μg/L [Santos et al. 2007]. Bisphenol A concentration in hazardous wastes may reach level 17 mg/L, and in water samples, 12 μg/L [Suzuki et al. 2004]. The progress of radiolytic processes was monitored not only in model aqueous solutions, as it was reported in earlier works [Homolok et al. 2011, Yu et al. 2013], but also in real environmental samples at the concentration level similar to that reported in some earlier environmental studies. For this purpose, mostly HPLC with UV–Vis detection was used, whereas for detailed identification of products of DCF radiolytic decomposition, the LC/MS/MS method was employed. The rate constants for radical reactions with DCF pulse radiolysis was determined using pulse radiolysis method with spectrophotometric detection.

## Experimental

### Chemicals

All chemicals applied were of the highest purity grade available and were used as received (Sigma, Aldrich), except acetonitrile, methanol, and potassium dihydrogen orthophosphate purchased from J.T. Baker, Poland. All target compounds of this study, namely diclofenac (DCF) (2-(2-(2,6-dichlorophenylamino) phenyl)acetic acid), ibuprofen (((RS)-2-(4-methyl-propyl)phenyl)propanoic acid), carbamazepine (5H-dibenzo[b,f]azepine-5-carboxamide), and bisphenol A were purchased from Sigma Aldrich, Poland. Solutions of analytes were prepared in deionized Milli-Q water.

Samples of river water were collected in Warsaw from the river Vistula in location prior to the outlet from the municipal wastewater treatment plant, whereas hospital wastewater was obtained from the local hospital.

### Irradiation facility

Within this study, aqueous solutions of investigated analytes or samples were γ-irradiated using ^60^Co source gamma chamber with a dose rate of 4.8 kGy/h. The samples were irradiated in closed 10-mL conical glass flasks. The dosimetry was carried out with Fricke solution.

### Analytical procedures

The reversed-phase HPLC determinations of DCF were carried out using Shimadzu LC-10 AT chromatograph with the diode array UV–Vis detector model SPD-M10A VP. The analytical column Phenomenex Luna C18 (2) 250 × 4.6 mm, 5 μm, and a guard column (Torrance, CA, USA) were used. The injected sample volume was 20 μL. In method A, with shorter total run time about 12 min, as eluent, the mixture of 0.2 M aqueous solution of formic acid and acetonitrile 40:60 at pH 4 was used. In method B, with total run about 60 min, as eluent, the mixture of 0.6 mM aqueous solution of KH_2_PO_4_, acetonitrile, and methanol in ratio 50:30:20 of pH 4 was used. In both methods, the UV detection was carried out at 220 nm.

The solid-phase preconcentration of analytes was carried with Oasis HLB columns (Waters), which were conditioned by flushing with 3 mL methanol and 3 mL water at 5 mL/min, then 500-mL sample was loaded. In the next steps, the bed was rinsed with 1 mL 5 % methanol, and analytes were eluted with 3 mL methanol.

The ion-chromatographic determinations of chloride were performed using chromatograph Dionex model 2000i/sp, equipped with an ASRS I electrochemical anion self-regenerating suppressor, conductivity detector, AG9HC guard column, and AS9HC analytical anion exchange column from Dionex. Mobile phase of the system was sodium carbonate and sodium bicarbonate with a flow rate of 1 mL/min.

LC/MS measurements were carried out using LC-Ion trap—time-of-flight MS system from Shimadzu. Chromatography was carried out using gradient elution with the following eluents A: 0.1 % formic acid and B: acetonitrile, 0.1 % formic acid. Flow rate: 0.5 mL/min. Gradient: 0–3 min: 30 % B, then from 3 to 10 min, 30–40 %, then 10–40 min, from 40 to 65 %, then 40–45 min, from 65 to 100 %, 45–46, 100 %, then 46–47, from 100 to 30 % (equilibration) and from 47–55 min, 30 % (initial mobile phase composition for equilibration).

The MS conditions were as follows: full-scan acquisition with polarity switching; mass range m/z 150–1000; ion trap accumulation time 50 ms

### Pulse radiolysis

The pulse radiolysis was carried out in the nanosecond pulse radiolysis facility based on the electron accelerator LAE 10 installed at the Institute of Nuclear Chemistry and Technology in Warsaw [Bobrowski 2005]. It allows to carry out pulse radiolysis experiments with pulse duration (4–10 ns, 100 ns), electron energy 10 MeV, and beam power 0.2 kW.

### Toxicity measurements

For the monitoring of toxicity changes of the irradiated solutions, two commercial- and one laboratory-made tests were employed. The Microtox® bacterial acute toxicity test based on measuring bioluminescence of *Vibrio fischeri* using M500Toxicity Analyzer from Azur Environment (Wokingham, UK). The Thamnotoxkit F™ microbiotest with crustacean *Thamnocephalus platyurus* was obtained from MicroBioTests Inc. (Mariakerke, Belgium). Spirotox acute toxicity test, based on the use of a very large ciliated protozoan *Spirostomum ambiguum*, was developed in the Department of Environmental Health Sciences, Medical University of Warsaw, where over four order of magnitude toxicity changes can be monitored for different groups of chemical compounds [Nałęcz-Jawecki et al. 1999].

## Results and discussion

### Optimization of analytical procedures employed

Due to a very wide use of diclofenac, hundreds of research papers were published on the development of various analytical methods for its determination in pharmaceutical preparations and physiological fluids, and for the monitoring of its residues in environmental samples. They include spectrophotometric, electrochemical, and especially commonly used the high-performance separation methods. GC/MS methods require a preliminary derivatization of polar DCF, e.g., [Farre et al. 2007, Shin et al. 2012]. In recent years, the most commonly used method is HPLC with different detections, mainly UV and mass spectrometry detection, which were also employed in this study. The HPLC-UV methods were employed in this work for the investigation of the decomposition yield of examined species in different conditions, whereas the LC/MS/MS for the identification of products of radiolytic decomposition of DCF.

**Fig. 1:**
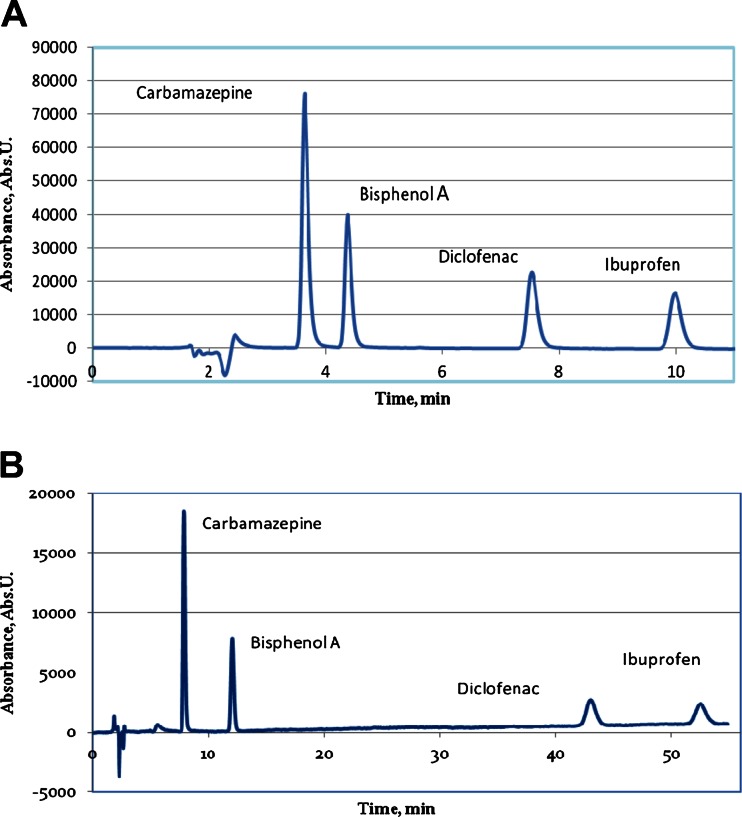
HPLC chromatograms recorded for standard mixtures of the determined analytes using two developed methods with isocratic elution. **a** Eluent composition: 40 % 0.2 M formic acid, 60 % acetonitrile, pH 4.0; injection 4 mg/L each analyte (method A). **b** Eluent composition: 50 % 0.6 mM KH_2_PO_4_, 30 % acetonitrile, 20 % methanol, pH 4.0; injection 2 mg/L each analyte (method B). In both cases: flow rate 1 mL/min, detection UV at 220 nm

A quick HPLC-UV method A was employed for the basic measurements in pure aqueous solutions of irradiated analytes, where a satisfactory baseline separation was obtained for all analytes in 12 min (Fig. 1a), with sufficient values of the limit of detection (LOD) (see Table 2) for the monitoring of decomposition of analytes at the initial concentration level of several milligrams per liter. This method, however, cannot be employed directly for the monitoring of radiolytic decomposition of analytes in natural waters of wastes due to a large signal of other constituents of natural samples, which are eluted with short retention times and they overlap signals for carbamazepine and bisphenol A, which were additionally monitored in some experiments besides DCF. The developed HPLC-UV method B exhibits also the baseline resolution of the examined solutes, allowing a significant reduction of the magnitude of overlapping signals from natural samples; however, it takes much longer time when isocratic elution is employed. For the routine monitoring of such a processes, a disadvantageous long running time can be substantially shortened by a careful optimization of the gradient elution.

**Table 2:** The limit of detection (LOD) values (μg/L) obtained in the employed HPLC-UV methods using 20-μL injection volume

Method	Diclofenac	Carbamazepine	Ibuprofen	Bisphenol A
Method A	26.3	7.90	35.0	15.1
Method A with 250-fold enrichment	0.11	0.03	0.14	0.06
Method B with 250-fold enrichment	2.30	0.30	2.96	0.66

For the application of those methods in micrograms per liter or sub-micrograms per liter concentration range, the additional step of the analyte preconcentration using a solid-phase extraction was also employed. It was carried out using commercial columns loaded with 60 mg Oasis HLB resin (Waters). In optimized conditions, the analytes were preconcentrated 250 times, with the use of 500-mL initial sample volume with pH adjusted to 8.0, and eluted with a satisfactory recovery using 2-mL methanol. The limits of detection (LOD) values for DCF, and also for three other analytes which were examined are listed in Table 2. They were considered as satisfactory for the investigation of the yield of radiolytic DCF decomposition using γ-irradiation in low micrograms per liter level.

### Optimization of the irradiation conditions

In dilute aqueous solutions (<0.1 M) the decomposition of organic pollutants by ionizing radiation is initiated by the primary products of water radiolysis ^•^OH, *e*_aq_^−^, and ^•^H, formed according to the following reaction [Buxton et al. 1988]:

1 $$ {\mathrm{H}}_2\mathrm{O}\ \underrightarrow{{}^{\mathrm{radiolysis}}}\kern0.5em {\left[0.28\right]}^{\bullet}\mathrm{O}\mathrm{H} + {\left[0.06\right]}^{\bullet}\mathrm{H} + \left[0.27\right]\ {e_{\mathrm{aq}}}^{-} + \left[0.05\right]\ {\mathrm{H}}_2 + \left[0.07\right]\ {\mathrm{H}}_2{\mathrm{O}}_2 + \left[0.27\right]\ {\mathrm{H}}^{+} $$

The numbers shown in brackets are *G* values (reported as radiolytic yield or radio-chemical yield) for each species (μM J^−1^) of adsorbed energy. Depending on employed chemical conditions during irradiation, and especially on the presence of purposely selected scavengers, one can adjust the concentration of selected predominating reactive species. For instance, in N_2_O-saturated solutions, as result of reactions (2) and (3):

2 $$ {e_{\mathrm{aq}}}^{-}\kern0.5em  + {\mathrm{N}}_2\mathrm{O} + {\mathrm{H}}_2\mathrm{O}\ \to \kern0.5em {\mathrm{N}}_2\kern0.5em +\kern0.5em \mathrm{O}{\mathrm{H}}^{\hbox{-}}\kern0.5em +{\kern0.5em }^{\bullet}\mathrm{O}\mathrm{H} $$

3 $$ {}^{\bullet}\mathrm{H} + {\mathrm{N}}_2\mathrm{O}\to {\kern0.5em }^{\bullet}\mathrm{O}\mathrm{H} + {\mathrm{N}}_2 $$

with the reaction rate constant values 9.1 × 10^9^ and 2.1 × 10^6^ M^−1^ s^−1^, respectively [Buxton et al. 1988], an increase of the concentration of hydroxyl radicals can be obtained, providing a strongly oxidizing conditions. It was reported that the total hydroxyl radical yield in N_2_O-saturated conditions is 0.59 μM J^−1^ [Buxton et al. 1995].

Similarly, the absence of reductive products of water radiolysis is observed when irradiated solutions are aerated, due to a fast reactions of those species with dissolved molecular oxygen (4, 5):

4 $$ {e}_{{\mathrm{aq}}^{\hbox{-} }} + {\mathrm{O}}_2\kern0.5em \to {{\mathrm{O}}_2}^{\bullet \hbox{-} } $$

5 $$ {}^{\bullet}\mathrm{H} + {\mathrm{O}}_2\kern0.5em \to \kern1em \mathrm{H}{{\mathrm{O}}_2}^{\bullet } $$

With the rate constant values 1.2 × 10^10^ and 1.9 × 10^10^ M^−1^ s^−1^, respectively [Buxton et al. 1988].

In case of the irradiation of deaerated solutions the addition of *t*-butanol to solution allows the removal of ^•^OH and ^•^H radicals from the solution as result of reactions (6) and (7):

6 $$ {}^{\bullet}\mathrm{O}\mathrm{H} + {\left(\mathrm{C}{\mathrm{H}}_3\right)}_3\mathrm{C}\mathrm{O}\mathrm{H}\to {}^{\bullet}\mathrm{C}{\mathrm{H}}_2\mathrm{C}{\left(\mathrm{C}{\mathrm{H}}_3\right)}_2\mathrm{O}\mathrm{H} + {\mathrm{H}}_2\mathrm{O} $$

7 $$ {}^{\bullet}\mathrm{H} + {\left(\mathrm{C}{\mathrm{H}}_3\right)}_3\mathrm{C}\mathrm{O}\mathrm{H}\kern0.75em \to {}^{\bullet}\mathrm{C}{\mathrm{H}}_2\mathrm{C}{\left(\mathrm{C}{\mathrm{H}}_3\right)}_2\mathrm{O}\mathrm{H} + {\mathrm{H}}_2 $$

with rate constant values 6.6 × 10^8^ and 1.7 × 10^5^ M^−1^ s^−1^, respectively [Buxton et al. 1988]. This allows utilizing the hydrated electrons, only, as a reactive species. In the same conditions, but at pH 2.0 or below, a fast reaction (8):

8 $$ {e}_{\mathrm{aq}}^{\hbox{-} } + {\mathrm{H}}^{+}\ \to {}^{\bullet}\mathrm{H} $$

takes place with the rate constant 2.3 × 10^10^ M^−1^ s^−1^ [Getoff 1996], providing conditions for the reaction with hydrogen radicals only.

Our preliminary studies on the irradiation of DCF were focused on the experimental determination of the yield of radiolytic decomposition in various conditions of irradiation, where different products of water radiolysis predominate [Trojanowicz et al. 2012] and the yield of decomposition was different in oxidative and reductive conditions due to different reactivity of predominating species under particular conditions. For the examined level of the initial DCF concentration 50 mg/L, it was showed that the DCF decomposition in aerated aqueous solution requires dose 4.0 kGy, while in the process carried out with the scavenging of the solvated electron by saturation of the irradiated solution with N_2_O, leading to substantial increase of hydroxyl radicals according to Eqs. (2) and (3), the required dose drops down to 1 kGy. The deaeration of irradiated solution by saturation with argon and irradiation in the presence of *tert*-butanol decreases yield of DCF irradiation only in the range of doses from 0.5 to 3 kGy, whereas above 3 kGy, it has no effect. This shows evidently, that the radiolytic degradation is more efficient in the oxidative conditions, and this is also confirmed by the calculated *G* values and evaluated rate constant values for reactions of DCF with hydroxyl radicals and hydrated electron, which are reported below.

**Fig. 2:**
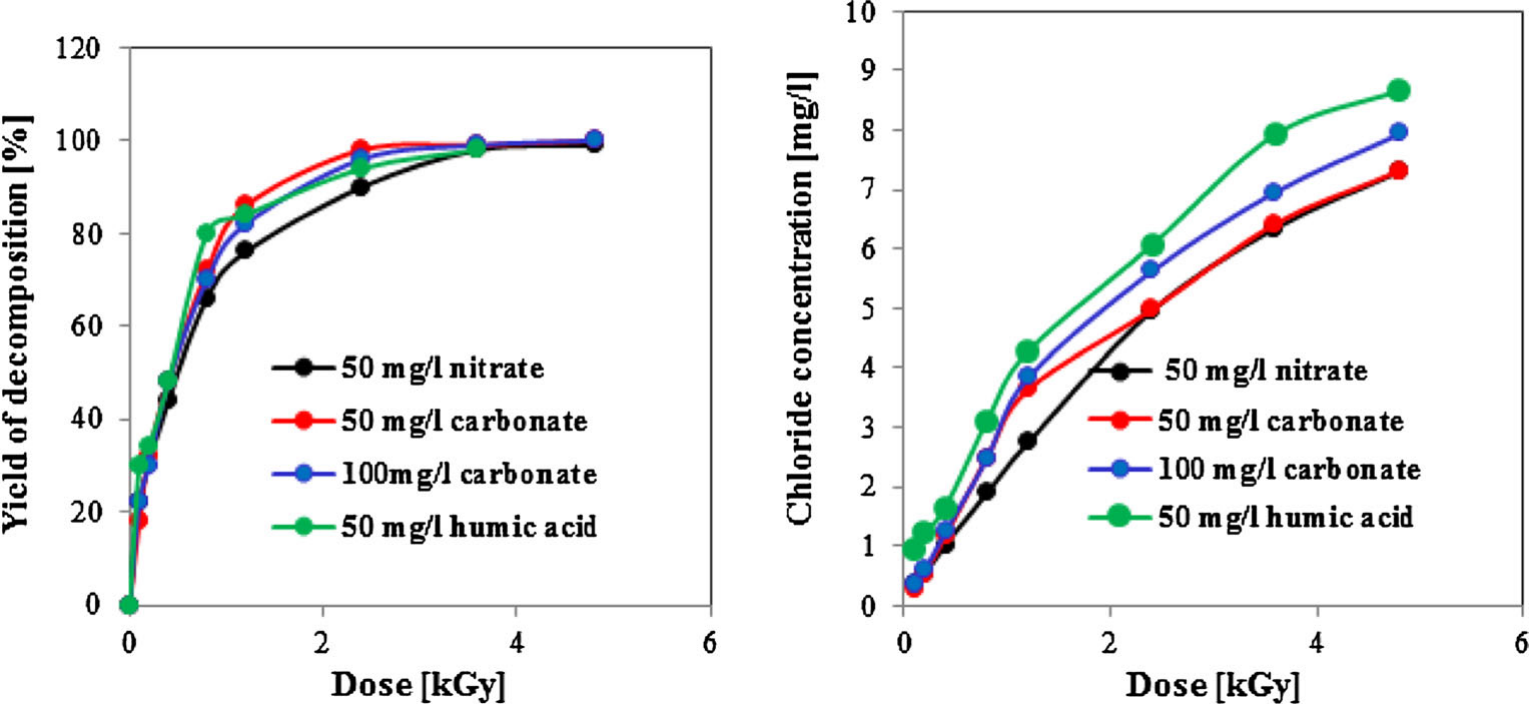
Chromatographic examination of the effect of selected scavengers of radicals on the **a** yield of DCF radiolytic decomposition and **b** on release of chloride in function of different absorbed dose. **a** Examined using the HPLC-UV method A. **b** Examined using the suppressed ion-chromatography with the conductivity detection. Gamma-irradiated aqueous solution of DCF 50 mg/L were saturated with air

The yield of radiolytic decomposition of organic pollutants in some cases may be affected by the presence of radical scavengers present in natural environmental samples. Most commonly, their effect results in lowering the yield of radiolytic decomposition of target pollutants; however, in some cases, also positive effects have been reported. In this study, which is focused on application of this method to natural waters and wastes, the optimized HPLC method A was employed to examine the effect of the presence of ^•^OH radical scavengers such as nitrate, carbonate, or humic acid on decomposition of DCF. As it is shown by data in Fig. 2, the yield of the DCF degradation at 50 mg/L level is not affected by the presence of those naturally occurring scavengers. Some effects, however, were observed for the yield of chloride released from DCF and its degradation products in different conditions of irradiation, where for instance, the presence of 50 mg/L carbonate or nitrate inhibits a complete dechlorination of organic products of the DCF decomposition. In contrary, the presence of the humic substances gives opposite effect, as it was also reported earlier for radiolytic decomposition of pesticide atrazine [Basfar et al. 2009].

**Fig. 3:**
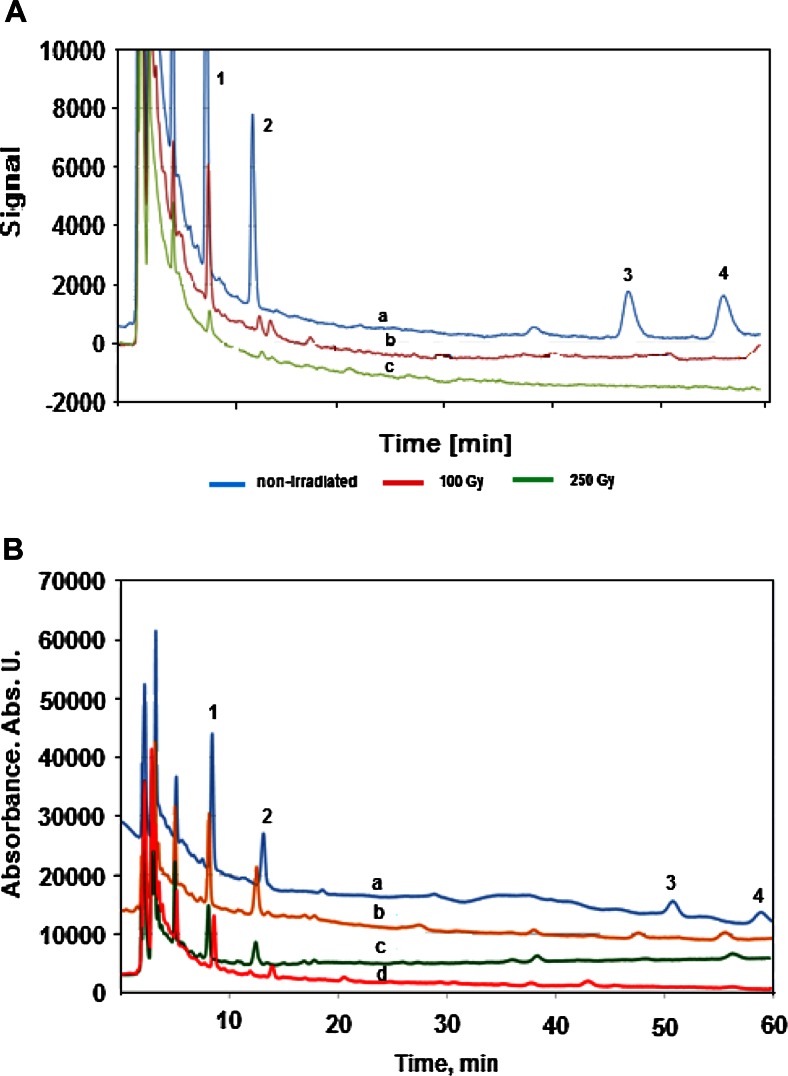
HPLC chromatograms obtained with method B and 250-fold SPE preconcentration for the aerated natural samples γ-irradiated with different doses. **a** Chromatograms of Vistula River water spiked with 10 μg/L concentration of each analyte, obtained for sample prior to the irradiation (*a*), after γ-irradiation with dose 100 Gy (*b*), and 250 Gy (*c*). **b** Chromatograms recorded in the same conditions for sample of wastewater from hospital spiked with 10 μg/L each analyte prior to γ-irradiation (*a*), after irradiation with 100 (*b*), 250 (*c*), and 500 (*d*) Gy dose. In both chromatograms: 1-carbamazepine, 2-bisphenol A, 3-DCF, 4-ibuprofen

The effectiveness of the radiolytic decomposition of DCF in pure aqueous solutions in different conditions of gamma irradiation was already examined and reported in several works cited in “Introduction.” It was showed that the most efficient decomposition occurs as result of reaction with hydroxyl radicals. In this study, the efficiency of decomposition was examined also in natural samples of a river water and hospital wastewater, which were spiked with 10 μg/L DCF, and also for comparison with three other common pollutants of natural waters, namely ibuprofen, carbamazepine, and bisphenol A. The irradiation was carried out in the oxidizing conditions in aerated samples, which are the simplest conditions for practical applications, which does not require any additional sample processing. At the same time, they are also favorable conditions from the point of view of a possible radical reactions. As it was already mentioned, in order to reduce the effect of matrix components, the HPLC measurements of irradiated solutions were carried out using method B with additional 250 times preconcentration with Oasis HLB column. As can be expected, and what is showed by chromatograms recorded at different absorbed doses (Fig. 3), the hospital effluent has a matrix exhibiting a larger content of constituents, which are retained on sorbent Oasis HLB, and they are then eluted in HPLC measurement with the retention time below 5 min. Certainly, many of those components can react with products of water radiolysis in competitive radical reactions, leading to decrease of the decomposition yield of examined analytes.

**Fig. 4:**
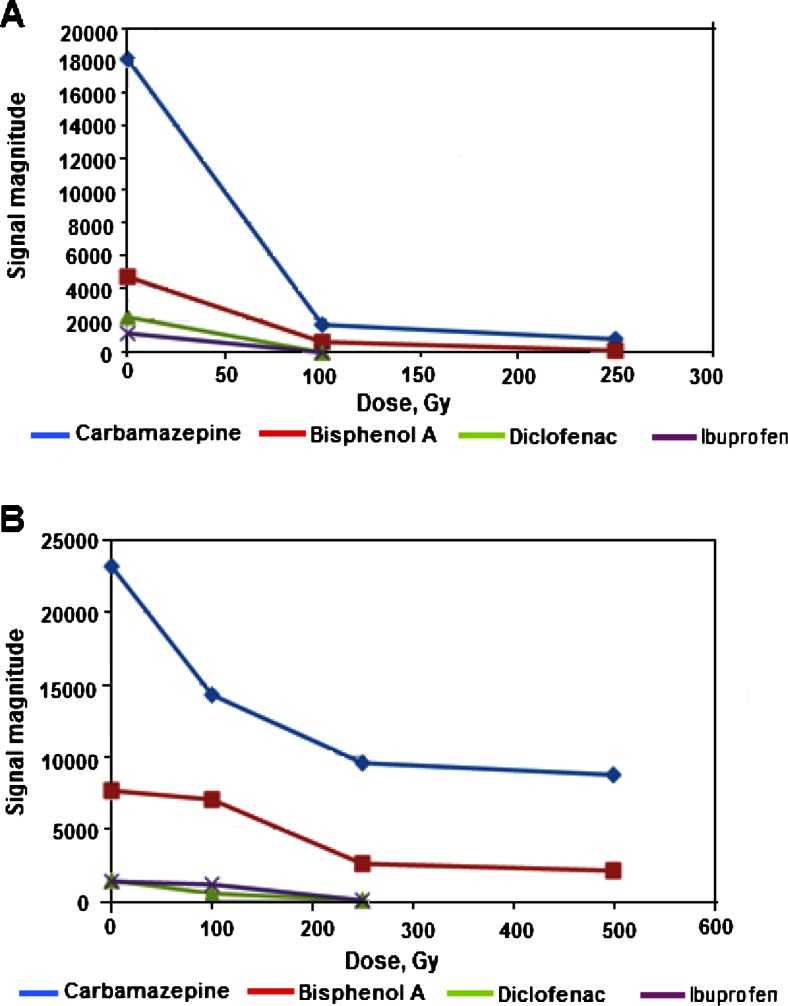
Comparison of the yield of γ-irradiation of investigated compounds in function of the absorbed doses, expressed by the signal magnitude for each analyte recorded in HPLC-UV measurements (method B) for samples spiked with 10 μg/L concentration each. **a** River water sample from Vistula. **b** Effluent from hospital

As shown in Fig. 3b, the irradiation of the hospital effluent with added 10 μg/L of each analyte was investigated with the use of absorbed doses up to 0.5 kGy. One can see, that only in case of diclofenac and ibuprofen, it was sufficient as the complete decomposition was observed at 0.25 kGy. The comparison of the effect of matrix type on the yield of decomposition of examined compounds is illustrated by the signal changes in recorded chromatograms for river water and hospital effluent samples (Fig. 4). The obtained results indicate that especially the decomposition of carbamazepine and bisphenol A requires much larger irradiation doses in case of the hospital effluent, than in river water samples. In case of bisphenol A, the application of 100 Gy absorbed dose allows the decomposition of 95 % in river water, while 70 % only in hospital effluent. For carbamazepine, those yields were 90 and 37 %, respectively. As far as diclofenac is concerned, for initial level of 10 μg/L used in spiked samples, as complete decomposition in river water was obtained for 100 Gy absorbed dose, while in the hospital effluent—250 Gy absorbed dose was needed.

Results of those experiments, performed close to a real content of residues, which can exist in the polluted environmental samples firmly show a very essential impact of the matrix composition on the yield of decomposition of pharmaceutical. The observed complete decomposition at absorbed dose level about 1 kGy indicates, that radiolytic AOP process may be a competitive method for the decomposition of pharmaceutical residues from waters and wastes compared to methods routinely used nowadays.

### Examination of toxicity changes

A very important supplement to analytical investigation of the efficiency of radiolytic decomposition of organic pollutants for environmental protection is the monitoring of toxicity changes in the irradiated solutions. This allows the determination of authentic environmental impact of applied processes with the use of different test organisms. An increased toxicity of DCF solutions was reported after photocatalytic treatment in the presence of TiO_2_ [Achilleos et al. 2010], as well as after (sono)photocatalytic processing [Michael et al. 2014], which was attributed to the formation of a variety of chloroderivatives of high toxicity.

**Fig. 5:**
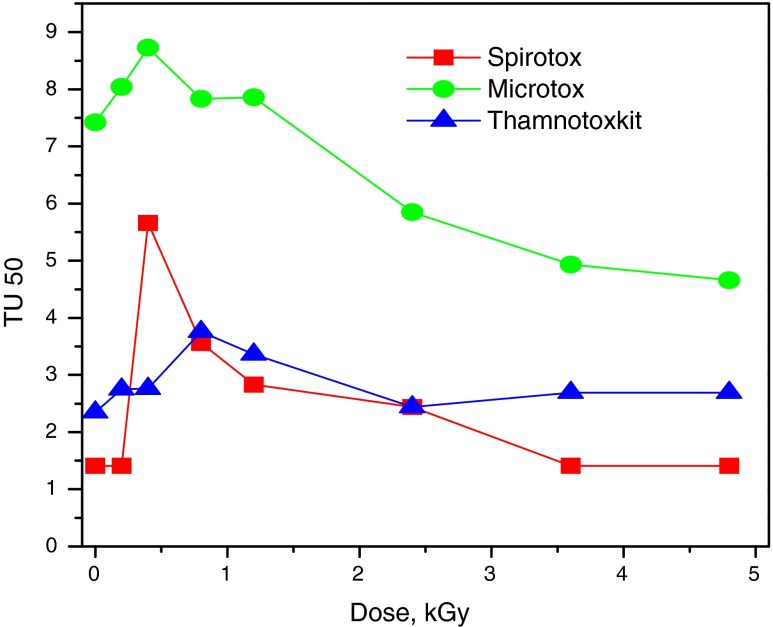
Toxicity changes observed with different toxicity tests at different absorbed doses for γ-irradiation of 50 mg/L aerated solution of diclofenac

The toxicity measurements in this work were carried out for pure aqueous solutions of diclofenac irradiated with different absorbed doses. Up to now, for this purpose, the Microtox test was used, only [Homolok et al. 2011, Yu et al. 2013], which is based on the use of the bioluminescent marine bacterium *V. fischeri* as the test organism. In order to get a wider scope of toxic properties of diclofenac and products of its radiolytic decomposition, besides the Microtox, two other tests were also employed, which are widely used in environmental toxicity studies. Spirotox is a test undertaken with a very large ciliated protozoan *S. ambiguum*, while Thamnotoxkit is a bioassay using larvae of the freshwater anostracan crustacean *T. platyurus* hatched from cysts. The toxicity studies were conducted for 50 mg/L DCF solutions irradiated in aerated solution with doses up to 5 kGy. With all employed tests, a certain increase of toxicity was observed for the applied doses in the range 0.5–0.8 kGy, where as it was shown earlier that about 50–60 % of DCF is decomposed [Trojanowicz et al. 2012]. This indicates a larger toxicity of formed transient products from decomposed DCF at low level of absorbed doses of radiation. As it was shown earlier, this effect quite often occurs when synthetic chemicals degrade in the environment [Boxal et al. 2004], and was also observed widely in numerous AOP processes reported in the literature as well, including photocatalytic DCF decomposition mentioned above [Achilleos et al. 2010; Michael et al. 2014]. At 3.5 kGy dose, where the complete decomposition of DCF is observed, 33 % decrease of Microtox toxicity was observed. Further decrease of toxicity of irradiated solutions can be expected at higher values of the absorbed dose, when the decomposition of produced chlorinated products are mineralized. On the other hand, practically no changes of toxicity was found in comparison to initial values prior to irradiation with two other employed tests (Fig. 5). This means in practice rather low toxicity of DCF for organisms employed in those two tests, and also an expected diversified environmental impact of DCF residues in waters and wastes on different organisms.

### Determination of decomposition products

In our preliminary investigation of products of the diclofenac radiolysis using the GC/MS method, only four main products were identified. Also, in earlier studies by other authors on radiolytic decomposition of DCF, only a few products of reactions of DCF with hydroxyl radicals and solvated electrons were identified [Liu et al. 2011, Homolok et al. 2011], while many more where detected, when LC/MS was employed, e.g., to the study of the decomposition of DCF by solar-driven photocatalysis [Aguera et al. 2005].

**Fig. 6:**
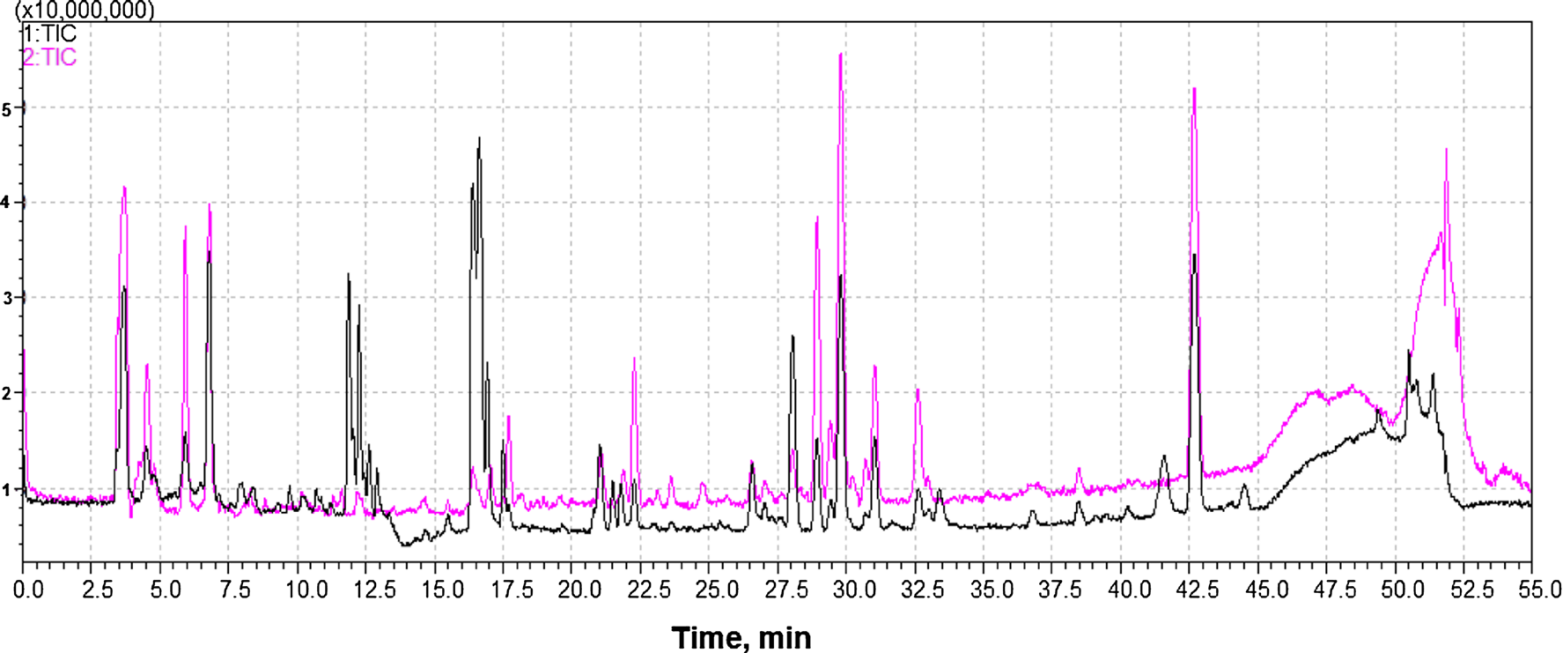
Total ion current chromatograms recorded in the LC-IT-TOF MS system with electro-spray ionization in negative (*1*) and positive (*2*) mode for aqueous aerated solution 50 mg/L DCF γ-irradiated with 3.6 kGy dose

**Fig. 7:**
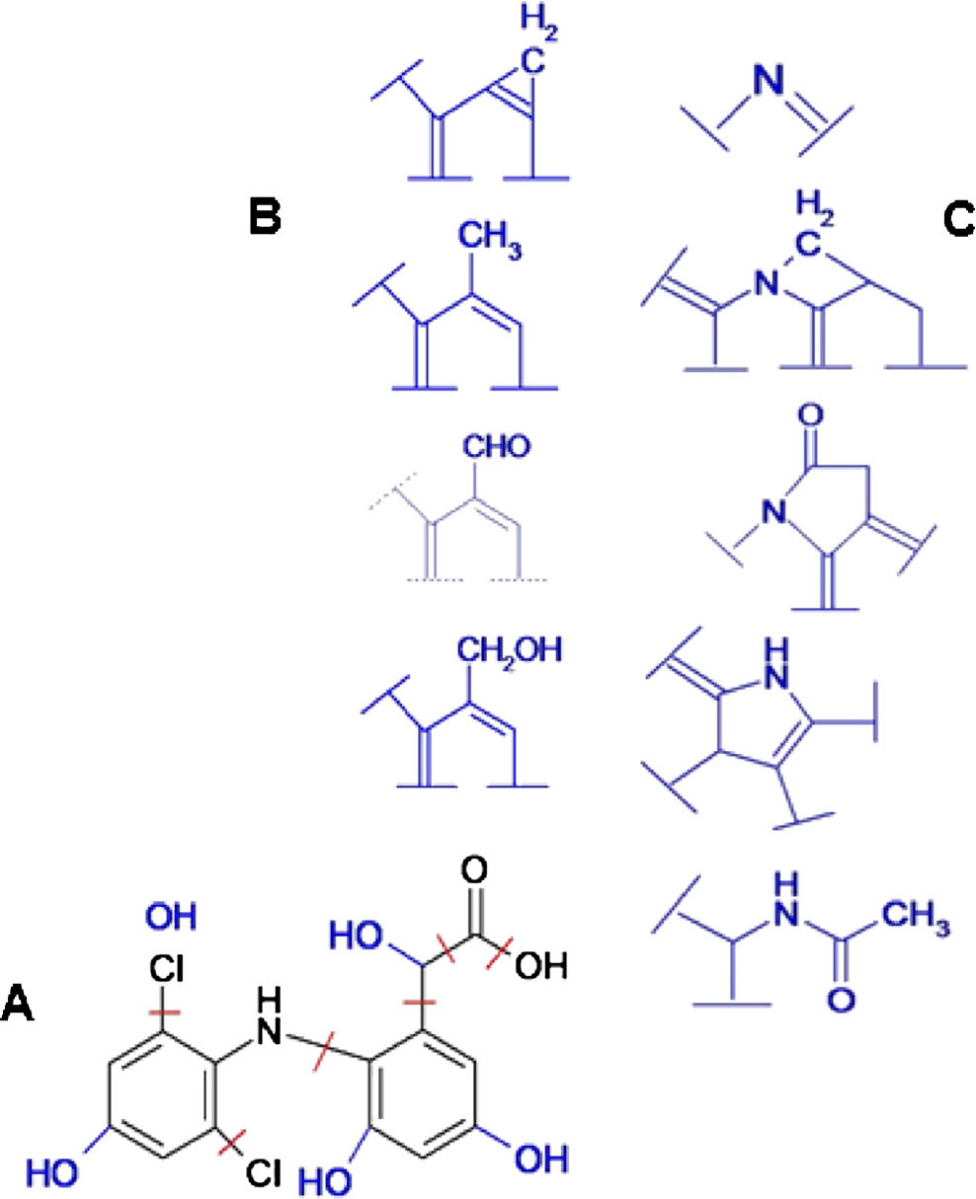
Schematic illustration of structural changes obtained in products of radiolytic degradation of diclofenac elucidated from obtained LC/MS data. **a** Breaking of bonds in DCF molecule showed by *red bars*, and positions of new substitute hydroxyl groups in DCF molecule marked in *blue*. **b** Structural changes of phenylacetic acid group of DCF molecule found among the products of DCF degradation. **c** Structural changes in part of DCF molecule surrounding amino group, which are deducted from identified products of DCF degradation

In these studies, using the HPLC analysis with mass spectrometry detection in the system with ion trap-time-of-flight analyzer (LC-IT-TOF MS), a much larger number of transient products were identified. The reported data concern γ-irradiation of aerated DCF solution at particular absorbed dose. As shown by the example total ion chromatograms, which were recorded with use of both modes of electro-spray ionization for 50 mg/L diclofenac solution irradiated with 3.6 kGy dose (where about 95 % decomposition of diclofenac occurs) ten different products of diclofenac radiolysis were detected at different concentration level (Fig. 6). Their partial identification is given by data listed in Table 3 (together with conditions of chromatographic separation and MS detection). This is the most detailed attempt reported so far compared to earlier approaches [Liu et al. 2011, Homolok et al. 2011, Kimura et al. 2012, Yu et al. 2013] to identify products of diclofenac radiolysis, and to illustrate the occurring processes. Based on the obtained LC/MS data, the structure of numerous products was elucidated and they are summarized schematically in Fig. 7. The identified products allow to conclude the location of breaking the bonds in diclofenac structure (red bars in Fig. 7A) and attachment of −OH groups in transient products showed by blue letters. Then, in Fig. 7B, modifications of phenyl acetic acid part of DCF molecule observed in identified products are showed. Similarly, in Fig. 7C the changes of amino group linking phenyl groups detected among the products of DCF radiolysis are showed. Because the detailed investigation of products was carried out for one particular example of DCF solution and one adsorbed dose only, the pathway of decomposition similar to reported for other degradation processes, e.g., [Aguera et al. 2005] cannot be suggested

**Table 3:** The main products of radiolytic decomposition of diclofenac detected by LC-IT-TOF MS in 50 mg/L aqueous solution of DCF irradiated with 3.6 kGy dose

Compound	RT (min)	Experimental m/z ([M + H]^+^)	Assigned elemental Composition [M]	DBE	Error (ppm)^*^	In-source CID fragments	Comments
Diclofenac (DCF)	42.70	296.0234	C_14_H_11_NO_2_Cl_2_	9	1.9	278; 214 …	
DCF-TP1	44.50	250.0184	C_13_H_9_NCl_2_	9	0.7	214/216	Neutral loss of formic acid (more apolar TP)
						215/217	
DCF-TP 2	44.00	248.0458	C_13_H_10_NOCl	9	6.0		
DCF-TP 3	43.87	230.0333	C_13_H_8_NOCl	10	14.9		Low int.
DCF-TP 4	41.58	214.0394	C_13_H_8_NCl	10	11.3		
DCF-TP 5	41.43	278.0126	C_14_H_9_NOCl_2_	10	2.8		
DCF-TP 6	40.25	262.0577	C_14_H_9_NOCl	9	20		Dechlorination
DCF-TP 7	40.22	254.0066	C_12_H_9_NOCl	8	7.9		
DCF-TP 8	37.11	244.0464	C_14_H_10_NOCl	10	24.5		−OH and −Cl
DCF-TP 9	33.62	274.0237	C_14_H_8_NO_3_Cl	11	10.4		+OH and −Cl
DCF-TP 10	32.96	264.0414	C_13_H_10_NO_3_Cl	9	3.0	246 (loss H_2_O)	
DCF-TP 11	32.50	294.0053	C_14_H_9_NO_2_Cl_2_	10	10.3	245/247	
DCF-TP 12	31.50	258.0309	C_14_H_8_NO_2_Cl	11	2.8	230.03/232.03	−Cl + OH
						260.0/262.03	
						197	
DCF-TP 12	30.90	278.0573	C_14_H_12_NO_3_Cl	9	2.0	248 (loss H_2_O)	
DCF-TP 13	30.70	266.0093	C_13_H_8_NOCl_2_	9	15.4		
DCF-TP 14	30.70	258.0309	C_14_H_8_NO_2_Cl	11	2.8		
DCF-TP 15	30.20	298.0050	C_13_H_9_NO_3_Cl_2_	9	6.0		
DCF-TP 17	29.10	312.0156	C_14_H_11_NO_3_Cl_2_	9	10.3	Position isomer	DCF –OH
						Of DCF- TP16	
						[Check 246.030 coeluting frag?]	
DCF-TP 18	28.10	260.0452	C_14_H_10_NO_2_Cl	10	8.0	Frag @	Dechlorination
						232.05/234.05	
						196/197/198	
DCF-TP 19	27.60	278.0546	C_14_H_12_NO_3_Cl	9	11.7		
DCF-TP 20	27.30	246.0295	C_13_H_8_NO_2_Cl	10	8.7		-Cl –ClH_2_
DCF-TP 21	27.10	264.0398	C_13_H_10_NO_3_Cl	9	9.1		
DCF-TP 22	27.00	278.0567	C_13_H_10_NO_3_Cl	9	4.1	Water loss	
						(*m/z* 260)	
						Coeluting *m/z*	
						294.0037. Need to check MS/MS	
DCF-TP 23	26.40	260.0441	C_14_H_10_NO_2_Cl	10	12.3		Dechlorination
DCF-TP 24	24.70	280.0237	C_14_H_11_NOCl_2_	9	19.1	Water loss	Low int. (Check SPE vial)
DCF-TP 25	23.80	244.0517	C_14_H_10_NOCl	10	2.8		Low int.
DCF-TP 26	23.70	294.0500	C_14_H_12_NO_4_Cl	9	9.4	Water loss	To be confirmed Elemental comp
			C_15_H_13_NOCl_2_ (2nd option)				

HPLC conditions: Gradient elution with following eluents A: 0.1 % formic acid; B: acetonitrile, 0.1 % formic acid. Flow rate: 0.5 mL/min. Gradient: 0–3 min: 30 % B, then from 3 to 10 min, 30–40 %, then 10–40 min, from 40 to 65 %, then 40–45 min, from 65 to 100 %, 45–46, 100 %, then 46–47, from 100 to 30 % (equilibration) and from 47 to 55 min, 30 % (initial mobile phase composition for equilibration). IT-TOF MS conditions: full-scan acquisition with polarity switching; mass range: *m/z* 150–1000; ion trap accumulation time: 50 ms

### Pulse radiolysis study

**Fig. 8:**
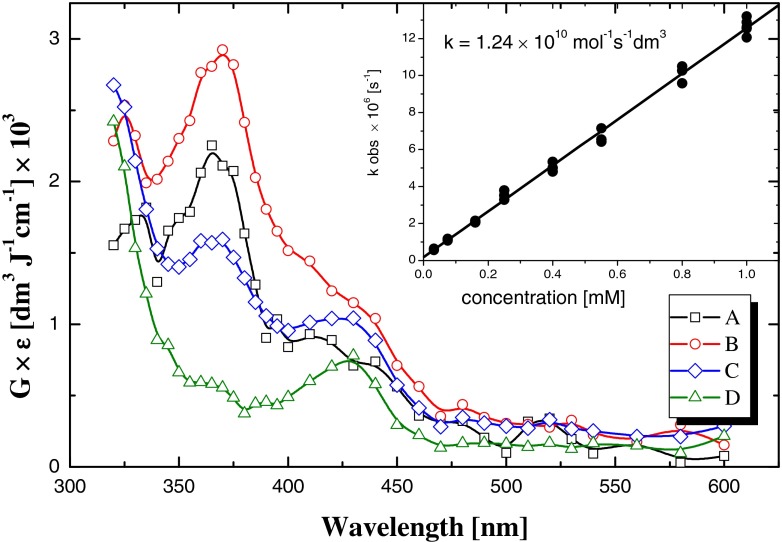
Absorption spectra recorded in N_2_O-saturated aqueous DCF solution in pulse radiolysis measurements. Spectra were collected after following time delays *squares* 0.12 μs; *circles* 0.8 μs; *diamonds* 16 μs, *triangles* 0.5 ms. *Inset* Stern-Volmer plot of the absorption growth at λ = 370 nm

As it was already mentioned above, in dilute aqueous solutions (below 0.1 M) radiolytic reactions of dissolved compounds involve radicals formed from radiolysis of water namely hydroxyl radical (^•^OH), and hydrated electron (*e*^−^_aq_) under the conditions in which these species predominate. In order to investigate the kinetics of ^•^OH reaction with diclofenac, the aqueous solution at pH 5.6 was saturated with N_2_O, which reacts with solvated electron and hydrogen atom according to reactions (2) and (3). The absorption spectra recorded during the oxidation of diclofenac by ^•^OH radical show three absorption bands with different intensity. The most intensive one is located at *λ*_max_ = 370 nm, and it grows up within <0.8 μs time domain (Fig. 8, curve B), with rate *k* = (1.30 ± 0.06) × 10^7^ s^−1^, and decay with rate *k* = (6.4 ± 0.5) × 10^4^ s^−1^. The second absorption band is located at shorter wavelength, and also is build up within microsecond time domain. The location of the second band is changing with the time, it starts at 335 nm (Fig. 8, curve A), after 0.8 μs, it moves to 325 nm (Fig. 8, curve B), and after 6 μs, is seen as a shoulder of the absorption band located at wavelength below 320 nm (start of measurement). This observation clearly indicates that at this region, more than one species respond for absorption, so its interpretation is ambiguous. The absorption spectra recorded 0.8 μs after the electron pulse indicates additional shoulder-shape absorption (Fig. 8, curve B), and with elapsed time, it revels the absorption band at 430 nm (Fig. 8, curves C and D).

The absorption spectrum recorded after end of OH-radical reaction with diclofenac (Fig. 8, curve B) can be attributed to primary product(s) of oxidation diclofenac by ^•^OH radicals. Probably it is adduct(s) of ^•^OH to aromatic rings of diclofenac according to the following scheme:
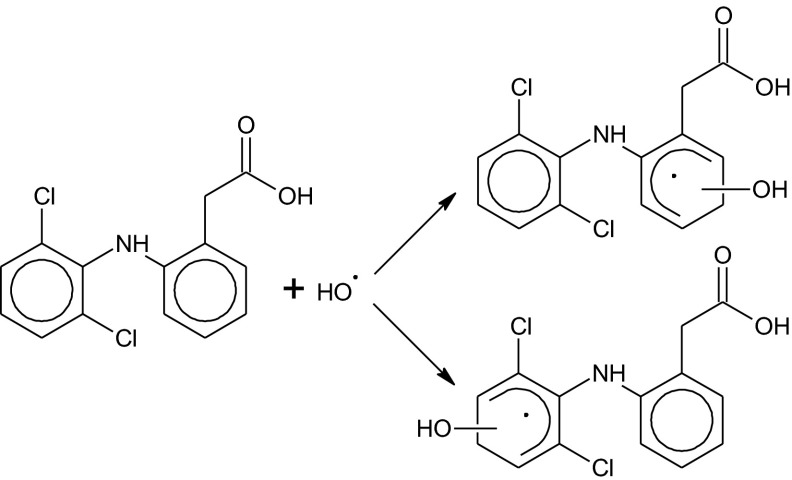


The overall rate constant of ^•^OH radicals reaction with diclofenac deducted from the Stern-Volmer plot of absorption build-up at λ_max_ = 370 nm is equal *k* = (1.24 ± 0.02) × 10^10^ M^−1^ s^−1^, (see inset in Fig. 8). This value is almost 40 % larger than reported earlier [Kimura et al. 2012, Yu et al. 2013], which was evaluated by the competition reaction method with phenol as the reference compound. In a longer time scale, we observed the decay of absorption band with maximum 370 nm, associated with the decay or transformations of primary products. The absorption of transient products of diclofenac oxidation recorded 0.5 ms after electron pulse (Fig. 8, curve D) reveals the absorption at 430 nm and a shoulder at a shorter wavelengths.

**Fig. 9:**
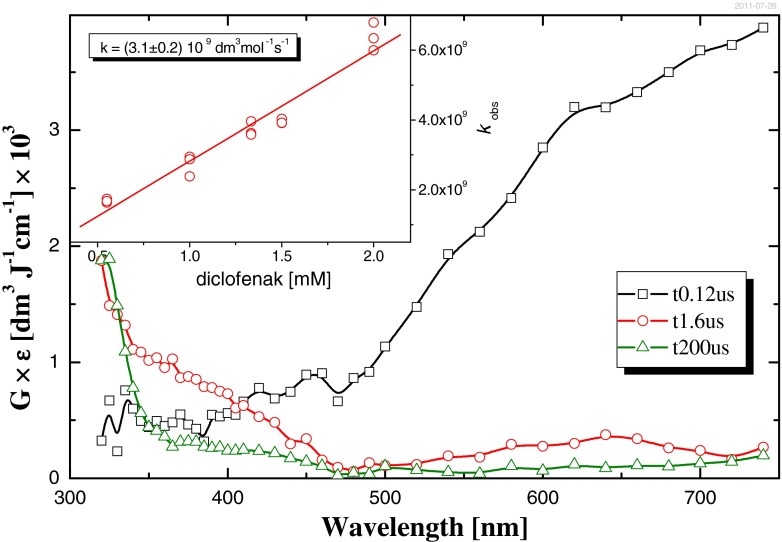
Absorption spectra recorded in Ar-saturated aqueous DCF solution containing 0.5 mM *tert*-butanol in pulse radiolysis measurements. Spectra taken after following time delays: *squares* 120 ns; *circles* 1.6 μs; *triangles* 200 μs. *Inset* Stern-Volmer plot of the absorption decay at λ = 700 nm

Investigation of diclofenac reaction with the solvated electron *e*^−^_aq_ has been performed in Ar-saturated aqueous solution. The 0.5 M tertiary butyl alcohol (*t*-BuOH) was added to scavenge the ^•^OH radical and hydrogen atom according to reactions (6) and (7). Almost three orders of magnitude excess of *t*-BuOH concentration comparing to DCF concentration is used in order to complete scavenge the mentioned above hydroxyl and hydrogen radicals. The absorption spectrum recorded 120 ns after the electron pulse (Fig. 9, curve A) exhibits the intensive absorption with maximum over 700 nm, assigned in the literature to hydrated electron [Boxal et al. 1976]. The spectrum recorded 1.6 μs after pulse (after decay of the hydrated electron see Fig. 9, curve B) exhibits absorption between 450 and 320 nm with no distinctive maximum. The kinetics of growth at 330 nm and decay at 700 nm are complementary (data not shown). We can attribute this absorption to the product of reaction *e*^−^_aq_ with diclofenac: diclofenac + *e*^−^_aq_ = > [diclofenac^•^]^−^. The overall rate constant of *e*^−^_aq_ with diclofenac deducted from the Stern-Volmer plot of absorption decay at 700 nm is equal *k* = (3.1 ± 0.2) × 10^9^ M^−1^ s^−1^ (see inset in Fig. 9). This value is two times larger, than that one reported by other authors [Yu et al. 2013]. The last spectrum (Fig. 9, curve C) recorded 200 μs after pulse shows the shoulder of absorption at 325 nm, infers that product of [diclofenac^•^]^−^ decay absorbs at shorter wavelength.

### Figures of merit of radiolytic decomposition

The kinetic characteristics of chemical reaction is determined by the order of reaction and the values of the reaction rate constants, however, the effectiveness of the irradiation processes is commonly expressed in *G* value, the value of dose constant and the dose required for 50 or 90 % decomposition of the irradiated solute [Mincher and Curry 2000]. *G* value (expressed in SI units in μM J^−1^), reported also as radiolytic or radiochemical yield, is defined as number of molecules formed or decomposed in the solution as result of absorbing 100 eV energy, and can be calculated at given absorbed dose using Eq. (9) [Kurucz et al. 1991]:

9 $$ G = \left[\left({C}_0\hbox{--}\ {C}_{\mathrm{D}}\right)\ {N}_{\mathrm{A}}\right]\ /\ D\ {K}_{\mathrm{f}} $$

where *C*_0_ is initial concentration of analyte [M], *C*_D_ is the concentration of analyte [M] after absorbing dose *D* [Gy], *N*_A_ is the Avogadro number 6.02 × 10^23^, and *K*_f_ is the conversion factor from Gy to 100 eV L^−1^, which is equal to 6.24 × 10^17^. The basic limitation of its usefulness is that it is usually concentration dependent, and therefore, very often rather the initial values *G*_0_ are calculated for the smallest applied dose near the beginning of the irradiation in function of absorbed dose. Although it is commonly reported in applied radiolysis, it is not considered as very useful for predicting the dose required to decompose the examined analyte.

Another factor, the observed dose constant *d*, determines the decomposition rate as function of the absorbed dose of radiation. It is calculated as the slope of the plot of –ln(*C*_D_/*C*_0_) vs. absorbed dose (Gy), and is considered as more accurate than *G*_0_ because it uses all the data from the irradiation procedure. The values of *d* are employed for determination of 90 % decomposition value as follows: *D*_0.90_ = (ln10)/*d* [Cooper et al. 1993].

The calculated values of those figures of merit *G*_o_, *d*, and *D*_0.9_ for DCF are compared in Table 4 with those obtained for three other examined analytes, and also with those calculated for radiolytic decomposition of a commonly used pesticide parathion (at given initial concentration values). In all cases, they concern the yield of radiolytic decomposition under oxidizing conditions, in air-saturated solutions without any additional pretreatment, where hydroxyl radicals are predominating reactive species, with simultaneous presence of O_2_^•−^ and HO_2_^•^ radicals according to Eqs. (4) and (5). For comparison, there are also values of rate constants shown for the reactions of a given analyte with the hydroxyl radicals (with bias regarding different published values). Those rate constant values are of the same order of magnitude for considered species, and the same can be said about *G*_o_ values, which were calculated for the smallest absorbed dose. Evidently, larger span of calculated values can be noticed for dose constant and *D*_0.9_ values, which are the most convincing quantities proving information about the yield of radiolytic decomposition process. Those values are similar for DCF, ibuprofen, and bisphenol A, while larger doses are required for decomposition of carbamazepine, and especially parathion. As shown, e.g., for polychlorinated biphenyls (Mincher et al. 1998], values of dose constant, and then also *D*_0.9_ are also concentration dependent, and must be evaluated for particular concentration level of pollutant.

**Table 4:** Figures of merit for the radiolytic decomposition of examined pharmaceuticals, pesticide parathion and bisphenol A using gamma radiation

Compound	Examined concentration, mg/L	Rate constant for reaction with ^•^OH, M^−1^ s^−1^	Radiolytic yield G_o_, μM J^−1^ (calculated at given absorbed dose, kGy)	Dose constant, kGy^−1^	*D*_0.9_ Dose required for 90 % decomposition, kGy
Diclofenac	10	12.4 × 10^9^ (this work)	0.147 (0.2)	6.02	0.38
Ibuprofen	10	6.7 to 10 × 10^9^	0.163 (0.2)	7.02	0.33
Carbamazepine	10	(2.0 to 9.7) × 10^9^	0.109 (0.2)	4.37	0.53
Bisphenol A	6.9	6.9 × 10^9^	0.141 (0.1)	7.19	0.32
Parathion	15	(4.2 to 9.7) × 10^9^	0.136 (0.2)	1.76	1.31

## Conclusions

Up to now, all data referring degradation diclofenac using ionizing radiation were obtained in pure aqueous solutions [Liu et al. 2011, Homolok et al. 2011, Yu et al. 2013], while in this study, the yield of degradation was evaluated also in samples of river water and hospital waste. Present studies were focused mostly on the radiolytic decomposition of diclofenac, but in measurements in real samples also additionally carbamazepine, ibuprofen also industrial pollutant bisphenol A were examined. Compared to previous studies, the conducted measurements in real samples were carried at concentration levels, close to those reported earlier in environmental samples. For DCF using the pulse-radiolysis method, the rate constants for reactions with hydroxyl radical (1.24 ± 0.02) × 10^10^ M^−1^ s^−1^ and solvated electron (3.1 ± 0.2) × 10^9^ M^−1^ s^−1^ were determined, showing that both the oxidative and reductive processes in radiolytic decomposition of DCF take place in irradiated diluted aqueous solutions of DCF. The conducting of DCF radiolytic decompositions, however, is more efficient in oxidative conditions. The radiolytic decomposition with required doses of gamma radiation from ^60^Co sources takes about 4 min, (when dose rate is 4.8 kGy/h) and compared to other developed methods are much quicker. The same processes carried out using electron beam irradiation could be practically considered as instantaneous.

In this study, also the influence of the absorbed dose magnitude (100 to 250 Gy) for the decomposition of selected other pollutants in river water was monitored. Under employed conditions, diclofenac and ibuprofen were completely destroyed with the use 100 Gy absorbed dose. Carbamazepine and bisphenol A, however, were still present even after irradiation with the absorbed dose 250 Gy. In the case of waste from hospital, the decomposition of carbamazepine and bisphenol A was more difficult. After 500 Gy, these compounds have not been successfully destroyed.

The essential complement of chemical control of decomposition pharmaceuticals during gamma decomposition is toxicity monitoring. In two earlier works [Homolk et al. 2011, Yu et al. 2013] for toxicity monitoring during the process used only bioluminescence test Microtox was employed. In our study, three different tests was applied, Microtox, Thamnotoxkit, and Spirotx. This allows to answer the question what is the authentic impact of degradation products on the environment.

Based on our study, it can be concluded that the HPLC method with UV detection and the appropriate preconcentration procedure can be used for the determination of pharmaceuticals and bisphenol A in water and wastewater at concentration level micrograms per liter. We expect that with the use of larger doses, a complete decomposition of more resistant compounds carbamazepine and bisphenol A can be also achieved.

## References

[CR1] Achilleos A, Hapeshi E, Xekoukoulotakis NP, Mantzvinos D, Fatta-Kassinos D (2010). Factors affecting diclofenac decomposition in water by UV-A/TiO_2_ photocatalysis. Chem Eng J.

[CR2] Agiunaco A, Beltran FJ, Garcia-Araya JF, Oropesa A (2012). Phlotocatalytic ozonation to remove the pharmaceutical diclofenac from water: influence of variables. Chem Eng J.

[CR3] Aguera A, Perez-Estrada LA, Ferrer I, Thurman EM, Malato S, Fernandez-Alba AR (2005). Application of time-of-flight mass spectrometry to the analysis of photo-transformation products of diclofenac in water under natural sunlight. J Mass Spectrom.

[CR4] Bae S, Kim D, Lee W (2013). Degradation of diclofenac by pyrite catalyzed Fenton oxidation. Appl Catal B Environ.

[CR5] Bartels P, von Tümpling JW (2007). Solar irradiation influence on the decomposition process of diclofenac in surface waters. Sci Total Environ.

[CR6] Basfar AA, Mohammed KA, Al-Abduly AJ, Al-Shahrani AA (2009). Radiolytic degradation of atrazine aqueous solution containing humic substances. Ecotox Environ Safety.

[CR7] Bobrowski K (2005). Free radicals in chemistry, biology and medicine: contribution of radiation chemistry. Nukleonika.

[CR8] Boxal ABA, Sinclair CJ, Fenner K, Kolpin D, Maund SJ (2004). When synthetic chemicals degrade in the environment. Environ Sci Technol.

[CR9] Buser HR, Poiger T, Müller MD (1998). Occurrence and fate of the pharmaceutical drug diclofenac in surface waters: rapid photodegradation in a lake. Environ Sci Technol.

[CR10] Buxton GV, Sdtuart CR (1995). Re-evaluation of the thiocyanate dosimeter for pulse radiolysis. J Chem Soc Farady Trans.

[CR11] Buxton G, Greenstock C, Helman WP, Ross AB (1988). Critical review of rate constants for reactions of hydrated electrons, hydrogen atoms and hydroxyl radicals (OH/O^−^) in aqueous solution. J Phys Chem Ref Data.

[CR12] Calza P, Sakkas VA, Medana C, Baiocchi C, Dimou A, Pelizzetti E, Albanis T (2006). Photocatalytic degradation study of diclofenac over aqueous TiO_2_ suspension. Appl Catal B Environ.

[CR13] Carlsson CJ, Alvan AK, Bergman G, Kuhler KT (2006). Are pharmaceuticals potent environmental pollutants? Part I: environmental risk assessments of selected active pharmaceutical ingredients. Sci Total Environ.

[CR14] Cooper JW, Cadavid E, Nickelsen GM, Lin K, Kurucz NC, Waite DT (1993). Removing THMs from drinking water using high-energy electron-beam irradiation. J Am Water Works Assoc.

[CR15] Daughton CG, Ternes TA (1999). Pharmaceuticals and personal care products in the environment. Environ Health Pers..

[CR16] Dobrin D, Bradu C, Magureanu M, Mandache NB, Parvulescu VI (2013). Degradation of diclofenac in water using a pulsed corona discharge. Chem Eng J.

[CR17] Farre M, Petrovic M, Barcelo D (2007). Recently developed GC/MS and LC/MS methods for determining NSAIDs in water samples. Anal Bioanal Chem.

[CR18] Fatta-Kassinos D, Meric S, Nikolaou A (2011). Pharmaceutical residues in environmental waters and wastewater: current state of knowledge and future research. Anal Bioanal Chem.

[CR19] Fent K, Weston AA, Caminada D (2006). Ecotoxicology of human pharmaceuticals. Aquat Toxicol.

[CR20] Ferrari B, Paxeus N, Lo Giudice R, Pollio A, Garric J (2003). Ecotoxicological impact of pharmaceuticals found in treated wastewaters: study of carbamazepine, clofibric acid, and diclofenac. Ecotox Environ Safety.

[CR21] Finkbeiner P, Franke M, Anschuetz F, Ignaszak A, Stelter M, Braeutigam P (2015). Sonoelectrochemical degradation of the anti-inflammatory drug diclofenac in water. Chem Eng J.

[CR22] Getoff N (1996). Radiation-induced degradation of water pollutants—state of the art. Radiat Phys Chem.

[CR23] Hartmann J, Bartekls P, Mau U, Witter M, von Tümpling W, Hofmann J, Nietz-Schmann E (2008). Degradation of the drug diclofenac in water by sonolysis in presence of catalysts. Chemosphere.

[CR24] Hofmann J, Freier U, Wecks M, Hohmann S (2007). Degradation of diclofenac in water by heterogeneous catalytic oxidation with H_2_O_2_. Appl Catal B Environ.

[CR25] Homolok R, Takacs E, Wojnarovits L (2011). Elimination of diclofenac from water using irradiation technology. Chemosphere.

[CR26] Houeto P, Carton A, Guerbet M, Mauclaire A-C, Gatignol C, Lechat P, Masset D (2012). Assessment of the health risk related to the presence of drug residues in water for human consumption: application to carbamazepine. Reg Toxicol Pharm.

[CR27] Ikehata K, Naghashkhar NJ, El-Din MG (2006). Degradation of aqueous pharmaceuticals by ozonation and advanced oxidation processes: a review. Ozone Sci Eng.

[CR28] Johnson AC, Dumont E, Williams RJ, Oldenkamp R, Cisowska I, Sumpter JP (2013). Do concentrations of ethinylestradiol, estradiol, and diclofenac in European rivers exceed propose EU environmental quality standards ?. Environ Sci Technol.

[CR29] Jones OA, Lester JN, Voulvoulis N (2005). Pharmaceuticals: a threat to drinking water?. Trends Biotechnol.

[CR30] Kimura A, Osawa AM, Taguchi M (2012). Decomposition of persistent pharmaceuticals in waste water by ionizing radiation. Radiat Phys Chem.

[CR31] Kumar A, Xagoraraki I (2010). Pharmaceuticals, personal care products and endocrine-disrupting chemicals in U.S. surface and finished drinking waters: a proposed ranking system. Sci Total Environ.

[CR32] Kümmerer K (Ed.) (2008) Pharmaceuticals in the environment: sources, fate, effects and risks. Springer-Verlag, Heidelberg Berlin

[CR33] Kurucz CN, Waite TD, Cooper WJ, Nickelsen MG, Lewnes J, Becker M (1991). High energy electron beam irradiation of water, wastewater and sludge. Advances in Nuclear Science and Technology.

[CR34] Langford KH, Thomas KV (2009). Determination of pharmaceutical compounds in hospital effluents and their contribution to wastewater treatment works. Environ Intern.

[CR35] Liu Q, Luo X, Zheng Z, Zheng B, Zhang J, Zhao Y, Yang X, Wang L (2011). Factors that have an effect on degradation of diclofenac in aqueous solution by gamma ray irradiation. Environ Sci Pollut Res.

[CR36] Luo Y, Guo W, Ngo HH, Nghiem LD, Hai FI, Zhang J, Liang S, Wang XC (2014). A review on the occurrence of micropollutants in the aquatic environment and their fate and removal during wastewater treatment. Sci Total Environ.

[CR37] Martinez C, Canle LM, Fernandez MI, Santaballa JA, Faria J (2011). Aqueous degradation of diclofenac by heterogeneous photocatalysis using nanostructured materials. Appl Catal B Environ.

[CR38] Michael I, Achilleos A, Lambropoulou D, Ososorio Torrens V, Perez S, Petrovic M, Barcelo D, Fatta-Kassinos D (2014). Proposed transformation pathway and evolution profile of diclofenac and ibuprofen transformation products during (sono)photocatalysis. Appl Catalysis B Env.

[CR39] Mincher BJ, Cooper WJ, Curry RD, O’Shea KE (1998). Radiolysis of polychlorinated biphenyls in nonpolar solvents. Environmental applications of ionizing radiation.

[CR40] Mincher BJ, Curry RD (2000). Considerations for choice of a kinetic fig. of merit in process radiation chemistry for waste treatment. Appl Radiat Isoto.

[CR41] Mompelat S, Le Bot B, Thomas O (2009). Occurrence and fate of pharmaceutical products, from source to drinking water. Environ Intern.

[CR42] Naddeo V, Belgiorno V, Ricco D, Kassinos D (2009). Ultrasonic degradation, mineralization and detoxification of diclofenac in waters: Optimization of operating parameters. Ultrason Sonochem.

[CR43] Naddeo V, Belgiorno V, Kassinos D, Mantzavinos D, Meric S (2010). Ultrasonic degradation, mineralization and detoxification of diclofenac in water: optimization of operating parameters. Ultrason Sonochem.

[CR44] Nałecz-Jawecki G, Sawicki J (1999). Spirotox—a new tool for testing the toxicity of volatile compounds. Chemisophere.

[CR45] Nie E, Yang M, Wang D, Yang X, Luo X, Zheng Z (2014). Degradation of diclofenac by ultrasonic irradiation: kinetic studies and degradation pathways. Chemosphere.

[CR46] Oaks JL, Gilbert M, Virani MZ, Watson RT, Meteyer CU, Rideout BA, Shivaprasad HL, Ahmed S, Chaudhry MJI, Arshad M, Mohamood S, Ali A, Khan AA (2004). Diclofenac residues as the cause of vulture population decline in Pakistan. Nature.

[CR47] OECD (2012) Pharmaceutical consumption. Chapter in “Health at a Glance Europe 2012. OECD Publishing. http://dx.doi.org/10.1787/9789264183896-38-en

[CR48] Perez-Estrada LA, Maldonado MI, Gernjak W, Agüra A, Fernandez-Alba AR, Ballesteros MM, Malato S (2005). Decomposition of diclofenac by solar driven photocatalysis at pilot plant scale. Catalysis Today.

[CR49] Perez-Estrada LA, Malato S, Gernjak W, Aguera A, Thurman EM, Ferrer I, Fernandez-Alba AR (2005). Photo-Fenton degradation of diclofenac: identification of main intermediates and degradation pathway. Environ Sci Technol.

[CR50] Ravina M, Campanella L, Kiwi J (2002). Accelerated mineralization of the drug diclofenac via Fenton reactions in a concentric photo-reactor. Water Res.

[CR51] Rizzo L, Meric S, Kassinos D, Guid AM, Russo F, Belgiorno V (2009). Degradation of diclofenac by TiO_2_ photocatalysis: UV absorbance kinetics and process evaluation through a set of toxicity bioassays. Water Res.

[CR52] Sanchez-Polo M, Lopez-Penalver J, Prados-Joya G, Ferro-Garcia MA, Rivera-Ytrilla J (2009). Gamma-irradiation of pharmaceutical compounds, nitroimidazoles, as a new alternative for water treatment. Water Res.

[CR53] Santos JL, Aparicio I, Alonso E (2007). Occurrence and risk assessment of pharmaceutically active compounds in wastewater treatment plants. A case study: Seville city (Spain). Environ Intern.

[CR54] Schulman LJ, Sargent EV, Numann BD, Faria EC, Dolan DG, Wargo JP (2002). A human health risk assessment of pharmaceuticals in the aquatic environment. Human Ecol Risk Ass.

[CR55] Schwaiger J, Ferling H, Mallow U, Wintermayr H, Negele RD (2004). Toxic effects of non-steroidal anti-inflammatory drug diclofenac Part I: histopathological alterations and bioaccumulation in rainbow trout. Aquat Toxicol.

[CR56] Shin H, Oh J (2012). Simultaneous determination of non-steroidal anti-inflammatory drugs in river water by gas chromatography–mass spectrometry. J Sep Sci.

[CR57] Snyder SA (2008). Occurrence, treatment, and toxicological relevance of EDCs and pharmaceuticals in water. Ozone Sci Technol.

[CR58] Song W, Cooper WJ, Mezyk J, Greaves SP, Peake BM (2008). Free radical destruction of β-blockers in aqueous solution. Environ Sci Technol.

[CR59] Stumm-Zollinger E, Fair GM (1965). Biodegradation of steroid hormones. J Water Poll Control Fed.

[CR60] Suzuki T, Nakagawa Y, Takano I, Yaguchi K, Yasuda K (2004). Environmental fate of bisphenol A and its biological metabolites in river water and their xeno-estrogenic activity. Environ Sci Technol.

[CR61] Tabak HH, Brunch RL (1970). Steroid hormones as water pollutants. Develop Ind Microbiol.

[CR62] Trojanowicz M, Bojanowska-Czajka A, Kciuk G, Bobrowski K, Gumiela M, Koc A, Nałęcz-Jawecki G, Torun M, Ozbay DS (2012). Application of ionizing radiation in decomposition of selected organic pollutants in waters. Eur Water.

[CR63] Vogna D, Marotta R, Napolitano A, Andreozzi R, d’Ischia M (2004). Advanced oxida-tion of the pharmaceutical drug diclofenac with UV/H_2_O_2_ and ozone. Water Res.

[CR64] Wang Y, Liu H, Liu G, Xie Y, Ni T (2015). Oxidation of diclofenac with chlorine dioxide in aquatic environments: influences of different nitrogenous species. Environ Sci Pollut Res.

[CR65] Yu H, Nie E, Xu J, Yan S, Cooper WJ, Song W (2013). Degradation of diclofenac by advanced oxidation and reduction process: kinetic studies, degradation pathways and toxicity assessments. Water Res.

[CR66] Zhang Y, Geissen SU, Gal C (2008). Carbamazepine and diclofenac: removal in waste-water treatment plants and occurrence in water bodies. Chemosphere.

[CR67] Zhao X, Hou Y, Liu H, Qiang Z, Qu J (2009). Electrooxidation of diclofenac at boron doped diamond: kinetics and mechanism. Electrochim Acta.

[CR68] Zwiener C, Frimmel FH (2000). Oxidative treatment of pharmaceuticals in water. Water Res.

